# Plasma cell-free DNA Droplet Digital PCR provides rapid and efficient infectious microbiology diagnosis for febrile haematological patients

**DOI:** 10.3389/fcimb.2025.1522426

**Published:** 2025-02-19

**Authors:** Ying Li, Jun Xiao, Lihui Xia, Xueqin Sun, Jia Li, Huili Bai

**Affiliations:** The Center for Clinical Molecular Medical Detection, The First Affiliated Hospital of Chongqing Medical University, Chongqing, China

**Keywords:** droplet digital PCR, cell-free DNA, infection, diagnostic performance, pathogen detection

## Abstract

**Background:**

Febrile haematological patients are at high risk for potential bloodstream infections, the rapid and accurate identification of pathogens is crucial for clinical diagnosis and treatment. Droplet Digital PCR (ddPCR) is a novel and ultra-sensitively molecular technique for the rapid detection of pathogens. We evaluated the ability of ddPCR to identify infectious etiologies to discuss the applicability of ddPCR in the diagnosis and treatment of infections for febrile haematological patients.

**Methods:**

This study enrolled and analyzed 89 ddPCR tests performed on 71 febrile haematological patients. We conducted a comparison between ddPCR results, blood culture (BC), and conventional microbiological testing (CMT). Additionally, we analyzed the correlation between ddPCR results and inflammatory factors, as well as their impact on antimicrobial therapy.

**Results:**

DdPCR detected 113 pathogens in 72 plasma samples, while CMT identified 39 pathogens in 32 plasma samples. The detection rate of bacteria and viruses using ddPCR was significantly higher than that of CMT (p <0.0001). The turnaround time (TAT) for pathogenic diagnosis was significantly shorter with ddPCR compared to CMT (p <0.0001). When we used the CMT as reference standard, the sensitivity and specificity of ddPCR were 93.8%, 26.3%, respectively. We observed a positive correlation between the ddPCR results and CRP, PCT and IL-6, and ddPCR (AUC=0.771) has better diagnostic performance. The anti-infective treatment strategies were adjusted for 30 patients based on the positive ddPCR results, with 86.7% (26/30) of these cases demonstrating effectiveness in the anti-infective treatment.

**Conclusion:**

DdPCR has the potential to enhance pathogen detection in febrile haematological patients by offering high sensitivity, rapid, precise results, it demonstrates better diagnostic performance compared to inflammatory factors and can contribute to the real-time clinical optimization of antimicrobial regimens, thereby enhancing the efficacy of anti-infective therapy.

## 1 Introduction

Malignant hematologic patients are at a heightened risk of bloodstream infections (BSIs) due to the immunocompromised state resulting from chemotherapy, hematopoietic stem cell transplantation, deep vein catheter placement, and post-chemotherapy myelosuppression (Liu et al., 2020; Wisplinghoff et al., 2003; Klastersky, 2004; Almyroudis et al., 2005; Averbuch et al., 2013; Schelenz et al., 2013; Zheng et al., 2017; Chen-Hua et al., 2018). Their inflammation-related clinical signs and symptoms may not be readily apparent, with fever being the most common indicator (Liu et al., 2020). Patients are commonly empirically treated with broad-spectrum antibiotics upon initial onset of fever (Liu et al., 2020; Klastersky, 2004; Averbuch et al., 2013), while there has been an increase in antibiotic resistance and complexity in recent years (Averbuch et al., 2013; Schelenz et al., 2013). Infections have a high mortality rate if the causative organisms are not promptly identified and treated with appropriate antimicrobial agents; some studies have reported a 7.1% to 42% mortality rate associated with bloodstream infections in patients with hematologic neoplasms (Zheng et al., 2017; Chen-Hua et al., 2018). Therefore, rapid and accurate pathogen diagnosis is crucial for optimizing anti-infective therapy and improving patient prognosis (Liu et al., 2020; Averbuch et al., 2013).

Currently, blood culture is still the gold standard for diagnosing bacterial or fungal infections in the bloodstream, but its sensitivity may be as low as 38% due to factors such as blood volume, technical challenges in specimen collection, prior antibiotic use, and contamination (Eichenberger Emily et al., 2022; Liu et al., 2024). Additionally, BC has a longer detection time ranging from 1-3 days (Tabak et al., 2018). Multiplex real-time polymerase chain reaction (RT-PCR) has recently become the most common molecular technique for rapidly detecting pathogens in whole blood (Opota et al., 2015). However, detection of pathogens in whole blood using RT-PCR remains challenging due to the low sensitivity resulting from a limited amount of pathogen DNA in the background of an excessive presence of human DNA (Gillis et al., 2024). Other technologies, such as pathogen macro-genome sequencing (mNGS), has the advantages of high throughput and screening for unknown pathogens, yet it still presents challenges in terms of operational complexity, high testing costs, and higher requirements for experimental conditions and bioinformatics analysis (Wu et al., 2022). Recently, ddPCR has been developed to provide technical advantages for addressing these challenges (Vogelstein and Kinzler, 1999; Hindson et al., 2011; Pinheiro et al., 2012). DdPCR is a novel and ultra-sensitively molecular technique for the rapid detection of pathogens. By dispensing PCR mix into tens of thousands of emulsified microdroplets, which serve as a miniature reaction chamber for PCR amplification, ddPCR demonstrates enhanced sensitivity and precision. This is attributed to its reduced susceptibility to PCR inhibitors and heightened sensitivity to microbial nucleic acid sequences. Moreover, this technology offers high reproducibility and provides absolute quantification without a standard curve (Wu et al., 2022).

DdPCR has emerged as a promising technology for the ultra-sensitive, fast, and accurate detection of pathogens in bloodstream infections (BSIs) (Salipante and Jerome, 2020; Chen et al., 2021). We aimed to provide rapid and accurate pathogen diagnosis for febrile haematological patients and optimize anti-infective therapy. The ddPCR panel, which contains 17 common bacteria and 5 common viruses, was designed to evaluate the clinical pathogen diagnostic efficacy and its value for antimicrobial management and prognostic therapy. This is the first evaluation of ddPCR for pathogen detection in hematologic patients as far as we know.

## 2 Materials and methods

### 2.1 Study design

**Figure 1:**
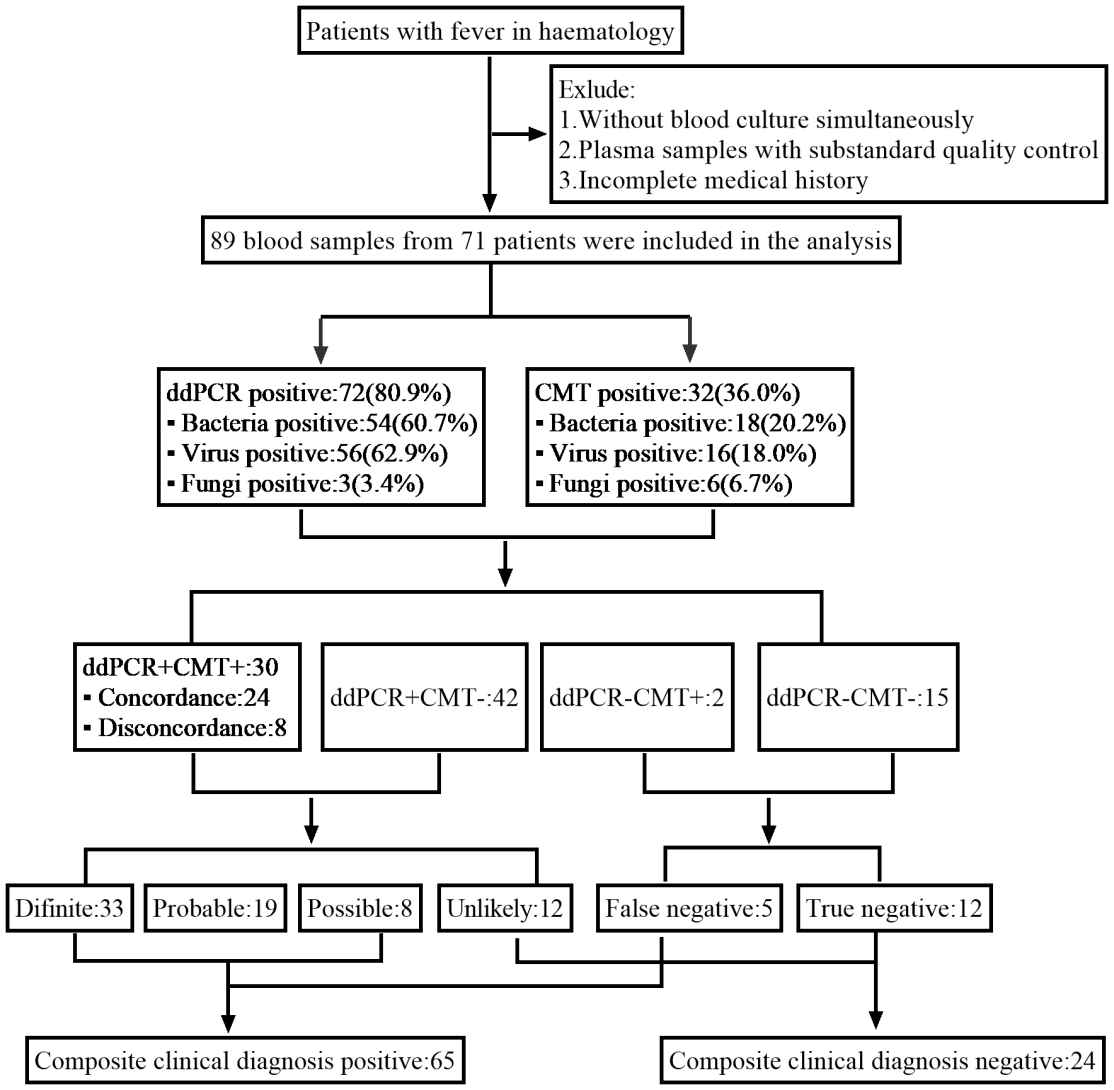
Schematic of the study profile.

The febrile patients information from the Department of Hematology of the First Affiliated Hospital of Chongqing Medical University from 23 June 2022 to 1 August 2023 were retrospectively collected in this study. Inclusion criteria were (1): hematologic diseases (2); meeting the criteria for fever: oral temperature ≥38.3°C (axillary temperature ≥38.0°C), or oral temperature ≥38.0°C (axillary temperature ≥37.7°C) for more than 1 hour. Exclusion criteria were (1): Without blood culture simultaneously (2); Plasma samples with substandard quality control (3); Incomplete medical records (**Figure 1**).

All patients received routine blood tests, CMT and ddPCR testing. They were retrospectively analyzed based on the demographic characteristics, underlying diseases, clinical signs and symptoms, radiologic manifestations, chemotherapy and transplant history, tracheal intubation, routine inflammation indicators results, ddPCR results, infection site, antimicrobial use, treatment response, and prognosis. The CMT chosen by the clinician included culture, smear, pharyngeal swabs, pathogen-specific antigens and serology assays (Liu et al., 2020; Klastersky, 2004; Averbuch et al., 2013),-b-D-glucan test (G test) and GM test, virus (EBV, CMV, HSV) PCR detection.

The clinical committee (includes 2 experienced clinicians and a laboratory technician) individually retrospectively made the diagnosis of different types of infection after a comprehensive consideration of those information. We compared the results of ddPCR with those of CMT to analyze the pathogen identification efficacy and clinical diagnostic efficacy of ddPCR, and evaluated the role of ddPCR for antimicrobial management and prognostic therapy.

### 2.2 Plasma cell-free DNA droplet digital PCR

5-10 ml of peripheral venous blood was collected from febrile patients with EDTA anticoagulation tubes, centrifuged at 1,600×g for 15 min at 4°C immediately after delivery to the laboratory. The cfDNA was extracted from 2 ml of the plasma supernatant by using the Auto-Pure nucleic acid purification Instrument (Allsheng, 10B) within about 30 minutes. The reaction mixture was passed through a micro-channel (Droplet Generator DG32) under the action of pressure to produce tens of thousands of water-in-oil emulsion droplets in 20 min. After PCR amplification for 50 min by Thermal Cycler (TC1), droplet counts and amplitudes were scanned and analyzed within about 30 min using a chip scanner CS5 and Gene PMS software (v1.0.4.220303). Positive controls were synthesized DNA fragments, and DNase-free water served as the negative control to eliminate external or reagent microbial contamination. The above operations all followed the manufacturer’s instructions, the whole testing process would take about 3 hours.

DdPCR detected common pathogenic microorganisms by labeling them with different fluorescence probe, included 13 Gram-negative bacteria (G^-^), 4 Gram-positive bacteria (G^+^), 5 virus and 1 fungi (**Supplementary Table S1** listed the range of pathogen detection for ddPCR). According to the manufacturers’ instructions, the target detection threshold for candida, streptococcus, and CoNS was 1.0 copies/ul, and the threshold for other pathogens was 0.5 copies/ul, the results higher than the threshold would define the ddPCR positive.

### 2.3 Clinical adjudication for ddPCR results

Based on a comprehensive analysis of laboratory data, radiological findings, and clinical symptom, the clinical committee independently categorized the results according to the likelihood that the pathogens identified by ddPCR would lead to an infectious febrile episode. The ddPCR results were categorized as definite, probable, possible, unlikely, and false or true negatives of the cases (The definitions summarized in **Supplementary Table S2**). Composite clinical diagnosis positive cases included the classifications of definite, probable, possible and false negatives, while the unlikely and true negatives cases were considered composite clinical diagnosis negative.

The clinical committee also evaluated the antimicrobial management of these patients and the effectiveness of anti-infective treatment. According to ddPCR results and clinically relevant decisions, the committee assessed the availability of ddPCR results for antimicrobial therapy, evaluating whether ddPCR results would contribute to timely changes in antimicrobial regimens, specifying whether antibiotic, antiviral, or antifungal agents would have been withdrawn or added. The efficacy of anti-infective treatment relied on the improvement of the patient’s clinical symptoms and the reduction of inflammatory markers (CRP, PCT, IL-6) within 7 days after receiving the ddPCR report, which was utilized to evaluate the impact of ddPCR results on treatment and prognosis.

### 2.4 Statistical analysis

Categorical variables were reported using frequencies and percentages, continuous variables were presented as mean ± standard deviation (SD) or the median. Differences in sensitivity, specificity, positive (PPV) and negative predictive values (NPV) between CMT and ddPCR were assessed using the Chi-square test. Kappa test was used for consistency of the two diagnostic results. Comparative analysis was conducted by the T test, Pearson’s correlation test, Mann–Whitney U test or Delong ROC test. P values <0.05 were considered significant. SPSS 21.0 (IBM Corp, Armonk, NY, USA) and GraphPad Prism 9.0 (GraphPad Software, San Diego, CA, USA) were employed for statistical analysis and to draw Figures.

## 3 Result

### 3.1 General characteristics

**Figure 2:**
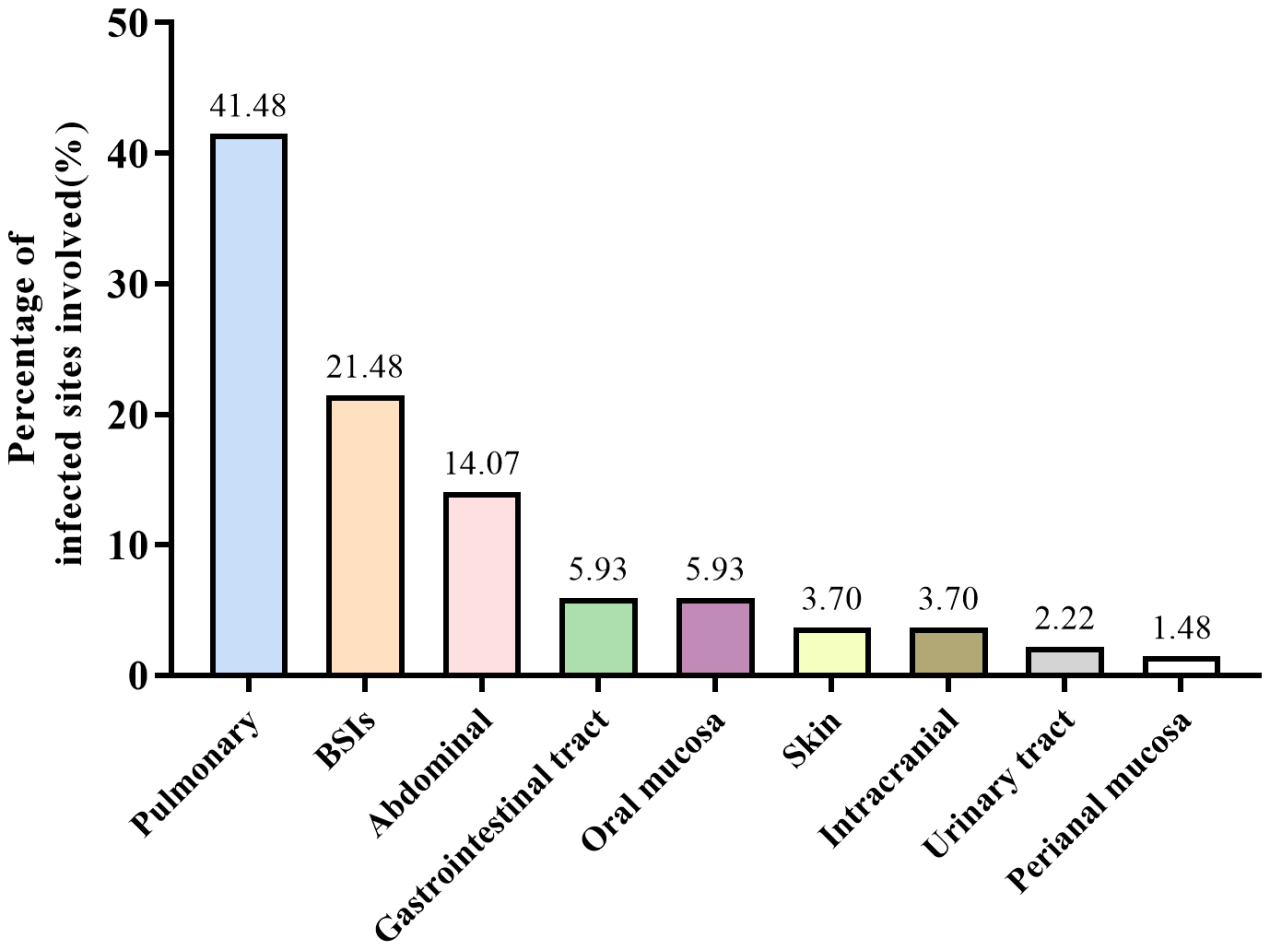
Distribution of infection site.

We screened all patients who underwent plasma ddPCR from June 2022 to August 2023 at the Department of Hematology, The First Affiliated Hospital of Chongqing Medical University. 71 patients were enrolled in this retrospective study based on the exclusion and inclusion criteria, a total of 89 blood samples were collected for ddPCR detection. 10 of patients had fever for 2 or more times during the hospitalization with one weeks apart from each fever at least. **Table 1** summarized the patient characteristics, including 54.93% males and 45.07% females, with a median age of 51 years. The most prevalent underlying diagnosis was acute myeloid leukemia (AML) in 29 cases (40.85%) and non-Hodgkin lymphoma (NHL) in 20 cases (28.17%). 63 (88.73%) patients had been treated with chemotherapy and 27 (38.03%) with hematopoietic stem cell transplant (HSCT). 52 (73.24%) of the patients had neutropenia and 68 (95.77%) had previous antibiotic exposure before ddPCR test. **Table 2** shows infection-related demographics, distribution of infection site, laboratory examination and other information. A total of 135 infections were recorded, with the most prevalent being 56 (41.48%) pulmonary infections and 29 (21.48%) bloodstream infections (BSIs) (**Figure 2**).

**Table 1:** Patients demographic and clinical characteristics in this study.

Samples characteristics	Total
Patients (n)	71
Gender, n (%)		
Male	39 (54.93%)
Female	32 (45.07%)
Age (y)		
Mean ± SD	49.28 ± 15.71
Median (range)	51 (16–86)
Diagnosis, n (%)		
AML	29 (40.85%)
NHL	20 (28.17%)
ALL	6 (8.45%)
MM	5 (7.04%)
MDS	4 (5.63%)
AA	3 (4.23%)
other	4 (5.63%)
With HLH, n (%)		
Yes	7 (9.86%)
No	64 (90.14%)
Chemotherapy history, n (%)		
Yes	63 (88.73%)
No	8 (11.27%)
Transplant history, n (%)		
Allo-HSCT	17 (23.94%)
Auto-HSCT	10 (14.08%)
No	44 (61.97%)
Neutropenia^a^, n (%)		
Yes	52 (73.24%)
No	19 (26.76%)
Previous antibiotic exposure^b^, n (%)		
Yes	68 (95.77%)
No	3 (4.23%)
Mechanical ventilation, n (%)		
Yes	9 (12.68%)
No	62 (87.32%)
Days of hospitalization, Mean ± SD	57.63 ± 59.17
28-day mortality, n (%)		
Yes	3 (4.23%)
No	68 (95.77%)

Neutropenia^a^: Absolute peripheral blood neutrophil count (ANC) <0.5×109/L or ANC <0.5×109/L expected after 48 hours; Previous antibiotic exposure^b^: the use of antibiotics at least 48 h within 3 days before the ddPCR test.

AML, acute myeloid leukemia; NHL, non-hodgkin lymphoma; ALL, acute lymphoblastic leukemia; MM, Multiple myeloma; MDS, myelodysplastic syndromes; AA, aplastic anemia; HLH, hemophagocytic lymphohistocytosis; Allo-HSCT, allogeneic hematopoietic stem cell transplantation; Auto-HSCT, autologous hematopoietic stem cell transplantation.

**Table 2:** Infection-related demographics and clinical characteristics in this study.

Clinical characteristics	Total
Fever samples (n)	89
Physical examination findings, Mean ± SD	
Body Temperature, °C	38.35 ± 1.08
Heart rate,/min	97.54 ± 22.23
Respiratory rate,/min	21.96 ± 5.92
Laboratory examination	
WBC, 10^9/L	
Mean ± SD	7.27 ± 26.35
Median (range)	0.38 (0.01-186.04)
ANC,10^9/L	
Mean ± SD	1.58 ± 3.32
Median (range)	0 (0-15.48)
PaCO_2_, mmHg	32.54 ± 7.81
CRP, mg/L	100.76 ± 85.86
PCT, ng/ml	6.78 ± 20.39
IL-2, IU/ml	2032.02 ± 2090.60
IL-6, pg/ml	485.54 ± 970.49
Infection site, n (%)	
Pulmonary	56 (41.48%)
BSIs	29 (21.48%)
Abdominal	19 (14.07%)
Gastrointestinal tract	8 (5.93%)
Oral mucosa	8 (5.93%)
Skin	5 (3.70%)
Intracranial	5 (3.70%)
Urinary tract	3 (2.22%)
Perianal mucosa	2 (1.48%)
Uncertain	2
Non-infection	4
COVID-19, n (%)	
Yes	6 (6.74%)
No	83 (93.26%)

WBC, white blood cell; ANC, absolute neutrophil count; PaCO_2_, partial pressure of carbon dioxide; CRP, C-reactive protein; PCT, procalcitonin; IL-2, interleukin-2; IL-6, iterleukin-6; COVID-19, corona virus disease 2019.

### 3.2 Distribution of pathogens

113 pathogens-positive results were detected from 72 plasma samples using ddPCR. The predominant bacterial organisms identified were Enterococcus spp., Escherichia coli, and Klebsiella, while the predominant non-bacterial organisms were EBV and CMV. 16 strains of positive pathogens were tested by BC, 23 positive pathogens results were determined by other CMT (6 by culture and 17 by qPCR), the most frequent pathogen were Escherichia coli, Klebsiella, EBV and CMV. **Table 3** summarized the pathogen category and amount in our study.

**Table 3:** The category and amount of pathogen detected in this study.

Pathogen category	Pathogen amount (n)			Total
	ddPCR	BC	Other CMT (sample, test)	
**Gram-Negative bacteria**	**23**	**11**	**4**	**38**
Escherichia coli	8	4	2 (sputum, culture; rectal swab, culture)	14
Klebsiella	8	3	1 (sputum, culture)	12
Acinetobacter baumannii	3	0	0	3
Pseudomonas aeruginosa	1	0	0	1
Hemolytic staphylococcus	1	0	0	1
Stenotrophomonas maltophilia	1	1	0	2
Burkholderia cepacia	1	0	0	1
Streptococcus mitis	0	1	0	1
Rod-shaped bacterium	0	1	0	1
Staphylococcus epidermidis	0	1	1 (sputum, culture)	2
**Gram-Positive bacteria**	**31**	**2**	**1**	**34**
Enterococcus spp.	18	1	0	19
Streptococcus spp.	7	0	1 (abdominal pus, qPCR)	8
Coagulase-negative staphylococci	3	0	0	3
Staphylococcus aureus	3	1	0	4
**Virus**	**56**	**/**	**16**	**72**
Epstein-Barr Virus	23	/	5 (plasma, qPCR)	28
Cytomegalovirus	20	/	6 (plasma, qPCR)	26
Herpes simplex virus 1	13	/	5 (plasma, qPCR)	18
**Fungi**	**3**	**3**	**2**	**8**
Candida	3	3	1 (oral secretions, culture)	7
Fumitremorgin C	0	0	1 (sputum, culture)	1
**Total**	**113**	**16**	**23**	**152**

The bold section shows the total for each pathogen category.

**Figure 3:**
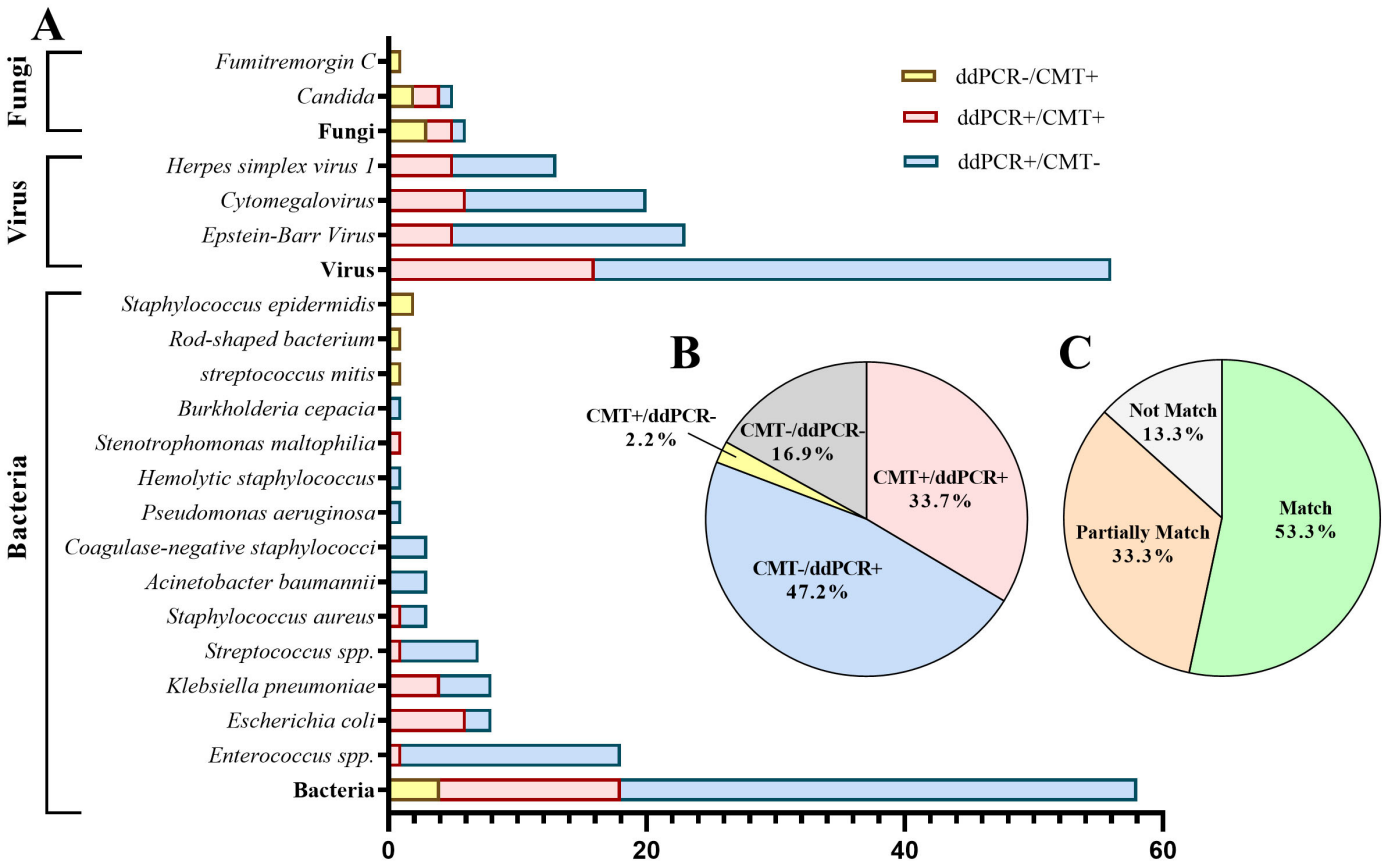
Distribution of pathogens identified in hematology patients by ddPCR versus CMT. **(A)** The distribution of pathogenic microorganisms detected by ddPCR and CMT. **(B)** Proportion of positive cases and negative cases detected by ddPCR and CMT. **(C)** Concordance between ddPCR and CMT in double-positive cases.

More pathogens were detected by ddPCR compared to CMT. Among the cases, 30 (33.7%) showed positive results for both methods, while 42 (47.2%) were positive with ddPCR but negative with CMT. Of the double-positive cases, 14 (51.9%) had completely matched results, 8 (29.6%) had partially matched results, and 5 (18.5%) had mismatched results (**Figure 3**).

Results of pathogen number detected by ddPCR and CMT were showed in **Table 4**. 53 cases were simple infections and 19 were mixed infections (with two or more pathogens) in ddPCR-positive cases. The detection rates of ddPCR for single infections and mixed pathogens were significantly higher than those of BC and CMT, with statistically significant differences.

**Table 4:** The comparison of pathogen species detected by ddPCR, BC, CMT.

Pathogens number	ddPCR	BC		CMT	
	(n, %)	(n, %)	P-value	(n, %)	P-value
0	17 (19.1)	73 (82.0)	/	57 (64.0)	/
1	53 (59.6)	16 (18.0)	<0.0001	24 (27.0)	0.001
≥2	19 (21.3)	0	<0.0001	8 (9.0)	0.036

### 3.3 Diagnostic performance

**Figure 4:**
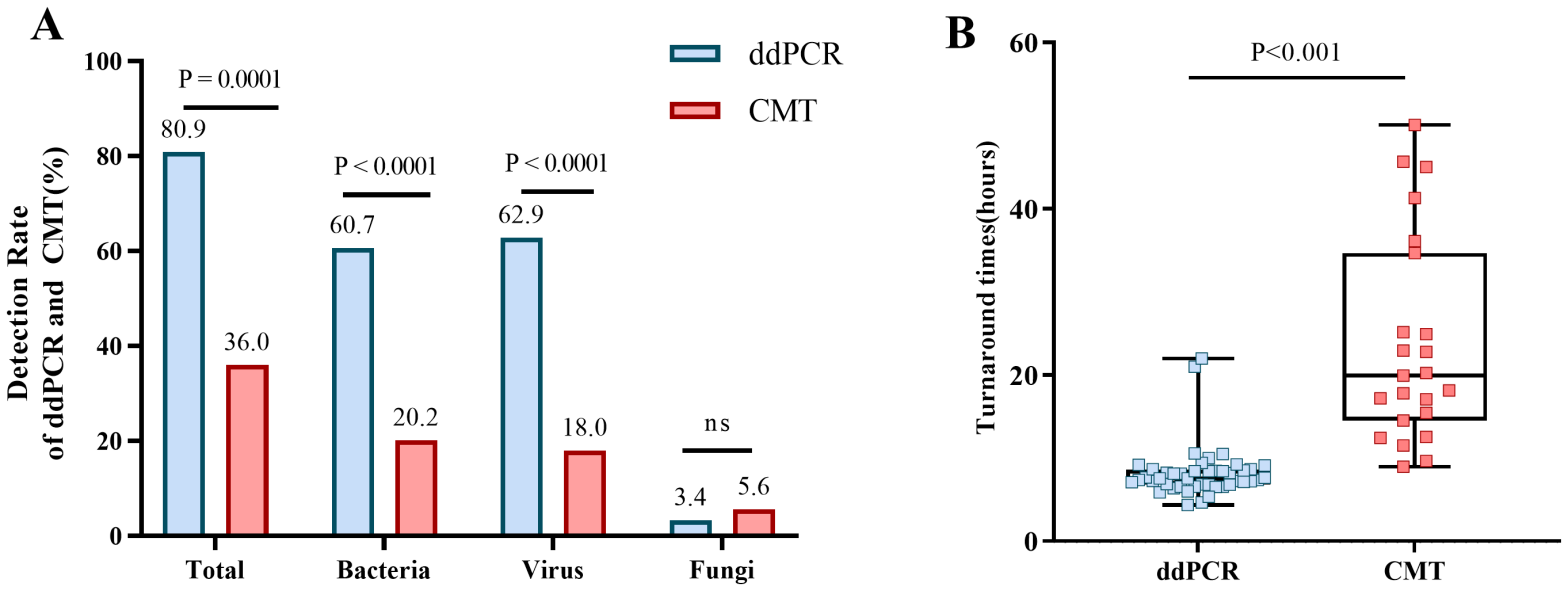
Positive rates for ddPCR and CMT pathogens and the comparion of TAT. **(A)** Positive rates of ddPCR and CMT for total, bacterial, viral, and fungal detection. **(B)** Comparion of TAT between ddPCR and CMT.

As shown in **Table 5**, the pathogen detection rate of ddPCR was significantly higher than that of BC (80.9% vs 18.0%, p< 0.0001) and CMT (80.9% vs 36.0%, p< 0.01). Additionally, the positive rate for bacteria and viruses of ddPCR was also higher than that of BC and CMT, with statistically significant differences. For fungi, the positively rate was no difference (**Figure 4A**).

**Table 5:** The comparison of detection rate for ddPCR, BC, CMT.

Pathogens category	ddPCR	BC		CMT	
	(n, %)	(n, %)	P-value	(n, %)	P-value
total	72 (80.9)	16 (18.0)	<0.0001	32 (36.0)	0.0001
bacteria	54 (60.7)	13 (14.6)	0.0001	18 (20.2)	<0.0001
virus	56 (62.9)	/	/	16 (18.0)	<0.0001
fungi	3 (3.4)	3 (3.4)	1.00	6 (6.7)	0.508

As displayed in **Table 6**, the diagnostic performance of the ddPCR was compared with BC, CMT and composite clinical diagnosis, respectively. When used blood cultures as a reference standard, the sensitivity, specificity, positive predictive value (PPV) and negative predictive value (NPV) of ddPCR were 87.5%, 57.5%, 31.1%, and 95.5%, respectively.

**Table 6:** The diagnostic performance of ddPCR results versus those of BC, CMT, and the composite clinical diagnosis.

	BC (+)	BC (–)	CMT (+)	CMT (–)	Composite clinical diagnosis (+)	Composite clinical diagnosis (-)
ddPCR (+)	14	31	30	42	60	12
ddPCR (-)	2	42	2	15	5	12
Sensitivity (%)	87.5		93.75		92.31	
Specificity (%)	57.53		26.32		50.00	
PPV (%)	31.11		41.67		83.33	
NPV (%)	95.45		88.24		70.59	
Kappa	0.264		0.157		0.466	
Accuracy (%)	62.92		50.56		80.90	

(+), positive; (-), negative; PPV, positive predictive value; NPV, negative predictive value.

Most importantly, the TAT required to determine the pathogenic diagnosis had significantly difference between ddPCR and CMT (7.56h vs 19.93h, P< 0.001) (**Figure 4B**). DdPCR yielded faster results in 80.9% (72/89) of all samples. In certain cases with positive BC, 75% (12/16) would have benefitted from an earlier diagnosis through ddPCR. DdPCR could also offer earlier definitive pathogen evidence in 50% (7/14) of patients with suspected infection by CMT.

### 3.4 Positive and negative concordance

Out of the 16 patients who tested positive for BC, 12 showed concordant results with ddPCR. In 3 cases, additional bacteria were detected, mainly involving gram-negative rods (GNR) such as E.coli (n=4), Klebsiella (n=3), and Stenotrophomonas maltophilia (n=1). Candida was detected in 2 cases, while Enterococcus spp. and Staphylococcus aureus were each found in 1 case. Two patients had discrepant results: Streptococcus mitis and Corynebacterium positive respectively in BC, but both were found to be positive for Enterococcus spp. by ddPCR. Another 2 patients were Candida aeruginosa and Epidermidis staphylococcus positive respectively in BC while ddPCR were negative (**Supplementary Table S3**). DdPCR was negative in 42 of 73 BC-negative cases. In 31 patients, ddPCR detected at least 1 bacterium or fungus undetectable by BC.

### 3.5 Correlative analysis and clinical performance comparison between ddPCR and inflammatory indicators

**Figure 5:**
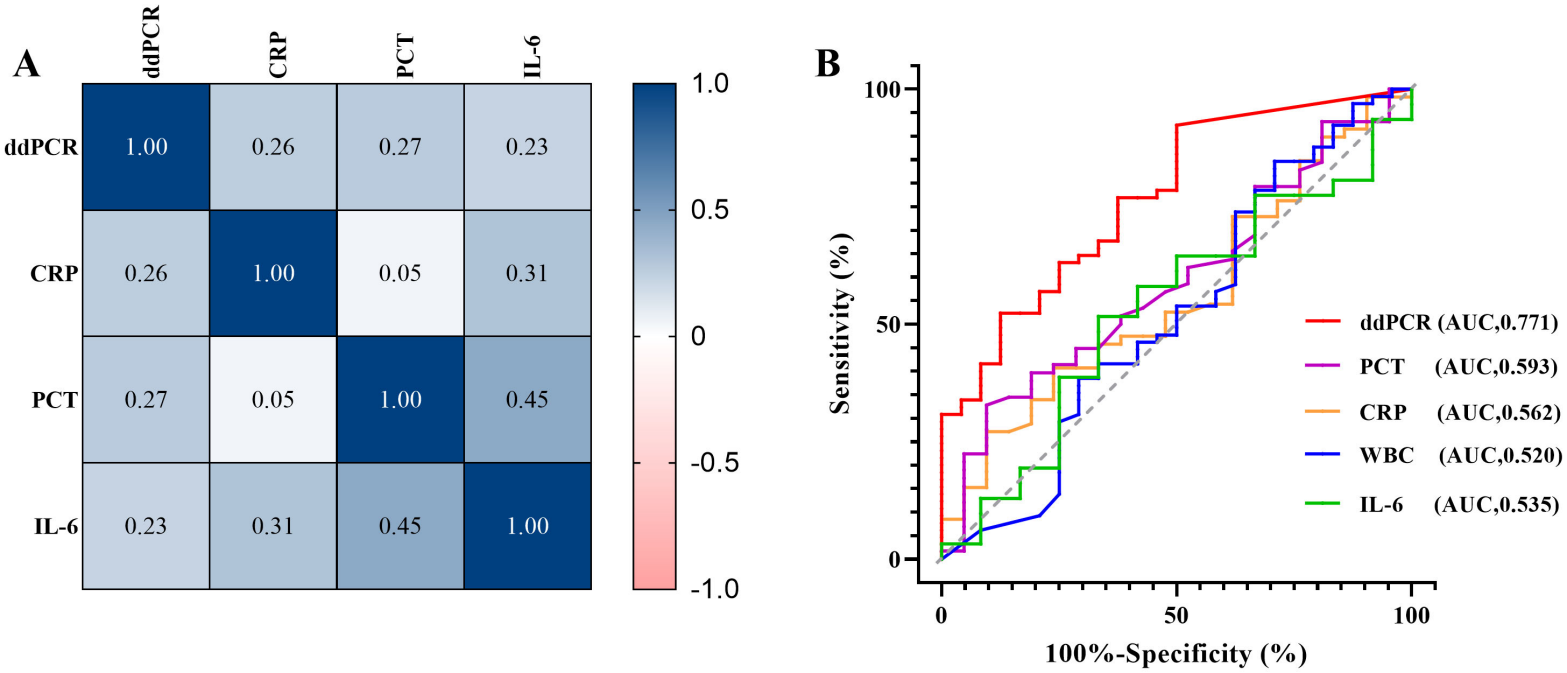
Correlative analysis and clinical performance comparison between ddPCR and inflammatory indicators. **(A)** Correlation between inflammatory markers (CRP, PCT, IL-6) and pathogen copy values detected by ddPCR. **(B)** The receiver operating characteristic curves (ROC) of ddPCR and inflammatory indicators (CRP, PCR, IL-6, WBC).

According to calculate Pearson correlation coefficient (R) between inflammatory markers (CRP, PCT, IL-6 and WBC) and plasma cfDNA detected by ddPCR, the log of plasma cfDNA copies was positively correlated with CRP, PCT and IL-6 (R> 0.2). There were less correlation between WBC and log of ddPCR copies (R=-0.095) (**Figure 5A**).

We also performed a ROC curve analysis using the clinical composite diagnosis as the reference standard, **Table 7** compared the area under the ROC curve (AUC) of the markers. The AUC of plasma cfDNA detected by ddPCR was 0.771 (95%CI 0.669 - 0.853, p<0.0001), demonstrating superior performance compared to other markers (**Figure 5B**).

**Table 7:** The comparison of AUC for markers.

Markers	AUC	95%CI	P-value
CRP	0.562	0.446-0.673	0.389
PCT	0.593	0.477-0.702	0.185
IL-6	0.535	0.377-0.688	0.729
WBC	0.520	0.412-0.627	0.789
Plasma cfDNA (ddPCR)	0.771	0.669 - 0.853	<0.0001

### 3.6 Antimicrobials management and clinical prognosis

**Figure 6:**
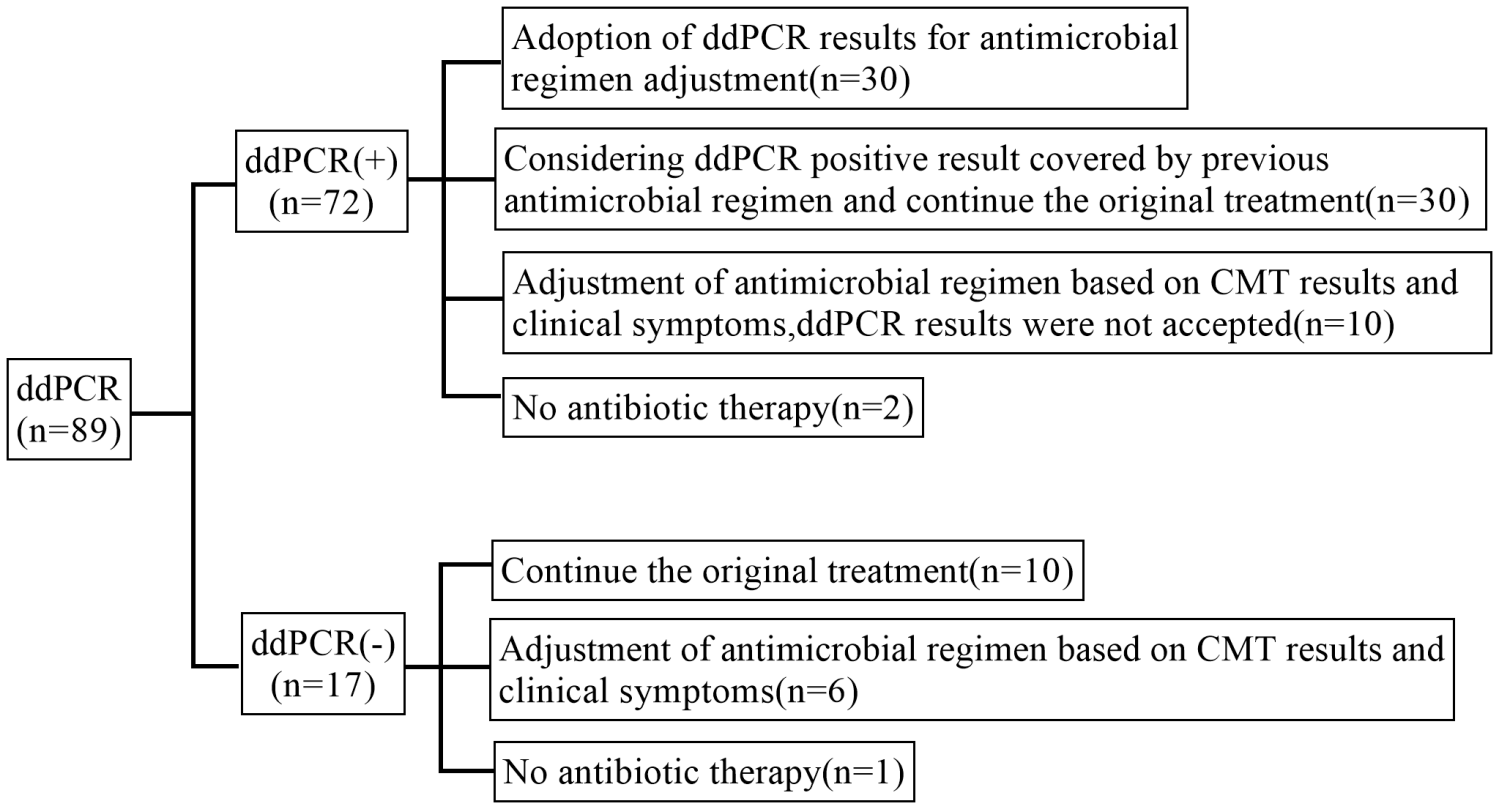
Post-test antibiotic adjustments.

In our study, 30 cases (41.7%) of ddPCR positive samples might be adopted by physicians and led to antimicrobial adjustments. 10 cases (11.2%) of ddPCR negative samples were continued the previous antimicrobial and antimicrobial were adjusted in 6 cases (6.74%). Besides, antimicrobials were adjusted based on CMT results and clinical symptoms in 16 cases (18.0%) whose ddPCR results were not accepted (**Figure 6**).

**Figure 7:**
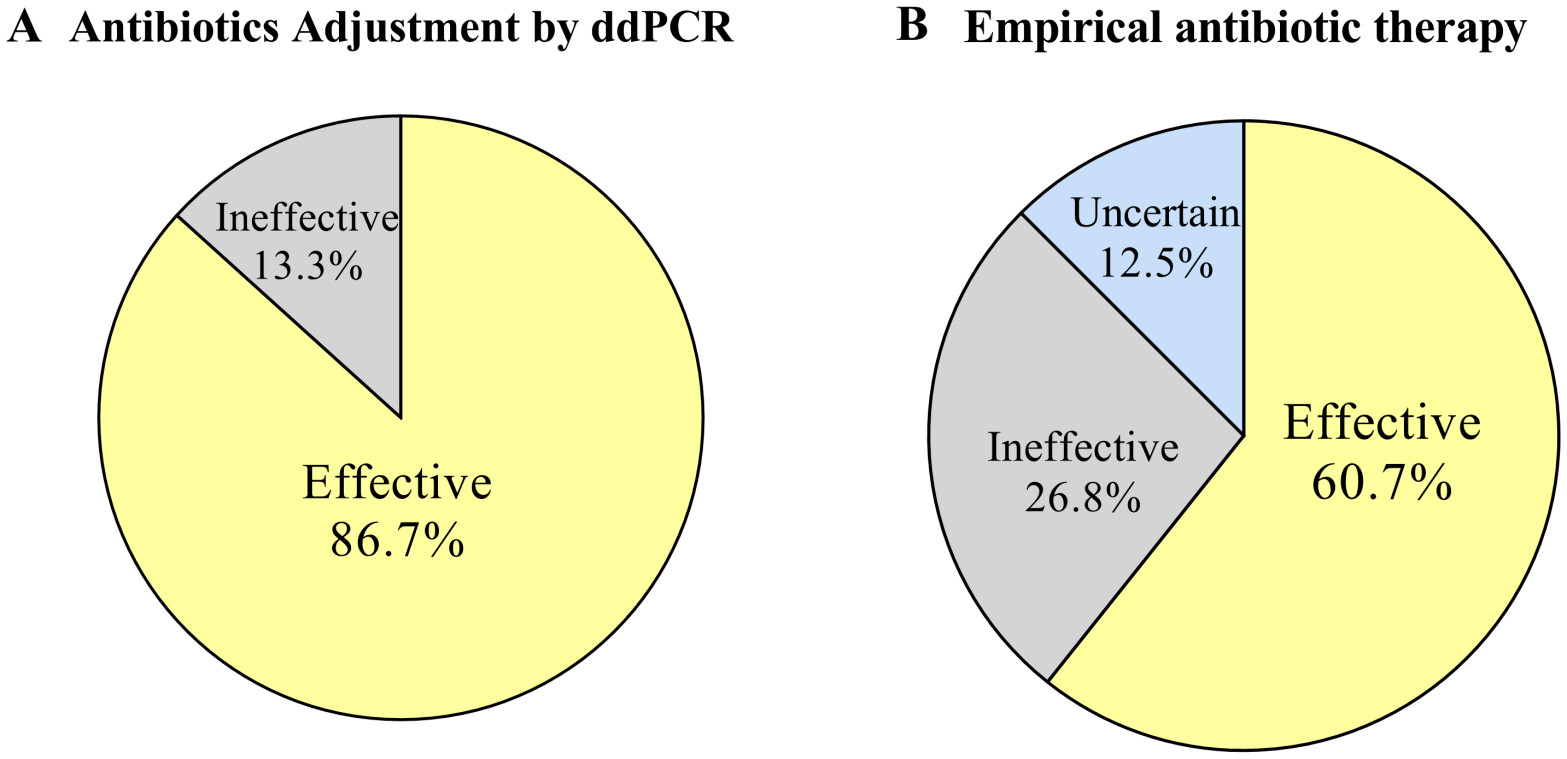
Effectiveness of antibiotic therapy. **(A)** Effectiveness of antibiotic adjustments therapy based on ddPCR results. **(B)** Effectiveness of empirical antibiotic therapy.

The committee evaluated the effectiveness of the antibiotic treatment regimen. We categorized cases without antibiotic adjustment and those adjusted for CMT or clinical symptoms as empirical antibiotic therapy, with at least 57.6% (34/59) of cases showing effective treatment, 25.4% (15/59) likely ineffective or not effective, and 16.9% (10/59) unable to determine effectiveness due to discharge or uncertain infection. The real-time availability of ddPCR results may lead to changes in antibiotics in 30 patients, with addition of antibacterials accounting for 10.1% (9/89), addition of antiviral and antifungal drugs followed in 6.7% (6/89) and 1.1% (1/89), respectively. De-escalation or discontinuation of antibacterial therapy would have been appropriate in 5.6% (5/89) of cases. Among the 30 antibiotic adjustment cases guided by ddPCR, 86.7% (26/30) of cases showed effective anti-infective treatment, 13.3% (4/30) of cases were likely ineffective or not effective (**Figure 7**).

## 4 Discussion

Malignant hematologic patients are at a heightened risk of developing bloodstream infections (BSIs) due to their compromised immune systems, and these infections have a high mortality rate if the causative organisms are not promptly identified and treated with appropriate antimicrobial agents (Averbuch et al., 2013). Rapid and precise pathogen identification is essential for optimizing anti-infective treatment and enhancing patient outcomes (Liu et al., 2020; Averbuch et al., 2013). In this retrospective study, we compared the results of ddPCR and CMT to analyze the pathogen identification and clinical diagnostic efficacy of ddPCR, and evaluated the value of ddPCR for antimicrobial management and prognostic therapy.

In our study, ddPCR demonstrated a significantly higher overall ability to detect pathogenic microorganisms compared to BC (80.9% vs 18.0%, p< 0.0001) and CMT (80.9% vs 30.6%, p< 0.01), with a wider variety and greater number of pathogens detected. Additionally, there were a relatively high sensitivity of 87.5% and a specificity of 57.5% for ddPCR compared with BC. Mixed infections are frequently observed in patients with haematological disorders (Chinese Society of Hematology et al., 2023). It is noteworthy that the detection rate of mixed infections by ddPCR was 21.3%, which was significantly higher than that of BC and CMT. Thus, ddPCR demonstrates clear advantages in pathogen detection based on our comparative analyses, potentially attributed to the following reasons: (і) The plasma cell-free DNA (cfDNA) of pathogenic microorganisms was chosen as detection target. As a new biomarker for liquid biopsy, circulating cell-free DNA (cfDNA) has been shown to be released early into plasma at low levels, with elevated concentrations observed in various pathological conditions, particularly in patients with infections (Jing et al., 2022). Additionally, cfDNA has a longer survival time in plasma and less affected by antibiotics (Han et al., 2020), which would be the eligible target for clinical diagnosis of infections; (ii) DdPCR has the advantage for ultra-sensitivity especially for nucleic acid analysis with low abundant targets, it can rapidly offer direct and independent quantification of cfDNA and give more precise and reproducible data without standard curves (Taylor et al., 2017; Blauwkamp et al., 2019). Compared to the traditional microbiological culture-based methods, ddPCR addressed time-consuming, low sensitivity, and the deviation of technician subjective judgment, etc. Consequently, ddPCR could complement the drawbacks of the traditional microbiological testing methods with a timely and accurate method for clinical diagnosis.

We observed that the positive detection rate of ddPCR for bacteria was significantly higher than that of CMT (48.3% vs 19.1%,p<0.0001). In our study, the predominant G^-^ flora detected in plasma were basically in line with the epidemiology previously reported (Yan et al., 2016; Cai et al., 2023). The predominant G^+^ flora detected by ddPCR were Enterococci, while Staphylococcus spp. was the most G^+^ flora detected by BC and reported in the other literature (Zhang et al., 2024). We have some hypotheses regarding these results: Enterococci have been increasingly considered causative agents of severe systemic infections, particularly in immunocompromised individuals. In the antibiotic era, Enterococci have been transformed from gut commensals to a leading cause of hospital infections, which could facilitate the colonization, pathogenesis, and persistence of various pathogens (Sangiorgio et al., 2024; Xu et al., 2024). According to our results, more than half of the Enterococcus positive cases suggested the infection may be mild due to the low copies, whereas it was undeniable that these case may be missed due to the traditional methods with low sensitivity. Hence, it’s significant to detect Enterococci using ultra-sensitive ddPCR in this easily overlooked infection. However, considering that enterococci are common gut commensals and hospital-contaminants (Fiore et al., 2019), the low copy results may be false positive due to the extreme sensitivity of ddPCR. Therefore, this aspect of the reports should be analyzed in conjunction with the clinical presentation. Additionally, the entire clinical picture must be considered when determining the clinical significance of a microbe detected by cfDNA, regardless of its concentrations (Blauwkamp et al., 2019).

In terms of viral detection, ddPCR performed better in positive detection rate compared to CMT with a significant difference (50.6% vs 14.6%,p<0.0001), and it was in general agreement with the spectrum of viral infections reported by Klara Obrova et al (Obrová et al., 2021). Screening for viral infections has gradually become part of routine diagnosis in recent years because of the high risk of infection of hematologic patients. Reportedly, viral infections of immunocompromised patients may result from a new infection or from reactivation of latent infections, which is consistent with development of immunosuppression, particularly in the most common human herpesviruses (HHVs) infection (Walton et al., 2014; Ong et al., 2017; Sehrawat et al., 2018). It was also may be responsible for a certain percentage of febrile episodes on hematologic neoplasms (Handous et al., 2020). Medium or high level viremia might be associated with febrile reactions. Therefore, screening for viral infections is essential to elucidate their potential role in patients with malignant disorders (Obrová et al., 2021). Andrew H. Walton et al. stated that serially tracking of viral load for a panel of latent viruses might be useful as indicators of the state of host immunity (Walton et al., 2014). Currently, qPCR is widely used in virus detection but has some shortcomings: (і) A standard curve is essential for the accurate absolute quantification of viral nucleic acid by qPCR, however, the production of a standard curve is significantly influenced by both inter-laboratory and day-to-day variability (Kojabad et al., 2021); (ii) qPCR is a semi-quantitative technique and cannot be detected when the viral load differences were less than one-half log (Hindson et al., 2013). As an absolute quantitative and ultra-sensitive technology, ddPCR performed better in sensitivity, precision, accuracy, and tolerance to PCR inhibitors than qPCR. Hence, ddPCR could be serving as a reliable tool for early warning and continuous monitoring viraemia in patients with pre-existing or new onset immunosuppressive disorders (Sedlak and Jerome, 2014; Jiang et al., 2024). We believe that the panel of ddPCR for detection of HHVs is well suited to the characteristics of viral infections in hematological patients.

Another interesting finding is that ddPCR pathogen copies showed positive correlation with inflammatory indicators such as PCT, CRP, and IL-6 in our study. Meanwhile, ROC analysis based on clinical composite diagnosis suggests that ddPCR has better diagnostic performance. Over the past years, PCT, CRP and IL-6 have been commonly used as indicators of inflammation in clinical infections, their elevated values are usually considered to be associated with an exacerbation of the infection, but they have some limitations. In clinical practice, there are wide variation in the threshold between markers (Carcò et al., 2022), and there are differences in underlying microbial etiologies, comorbidities, and antibiotic treatments (Aulin et al., 2021), these factors often affect the selection and credibility of inflammatory markers. Chun-Wei Wu et al. reported that PCT might be specific but not sensitive in differentiating severe bacterial infection from other systemic inflammation or viral infection (Svaldi et al., 2001; Wu et al., 2015). Previous studies have delineated that CRP remains at a high level during the anti-infection process and cannot predict antimicrobial efficacy. In addition, CRP has low specificity due to the disease severity affect in patients (Zheng et al., 2024). It has been reported that proliferative diseases of the hematopoietic system impact baseline CRP, such as lymphoma induces significantly higher initial CRP levels, while leukemia causes a moderate increase or no effect at all at the same time (Zheng et al., 2024). Evidence has confirmed that IL-6 cannot predict bacteremia due to the low sensitivity and PPV values (Zheng et al., 2024). Considering the insufficient inflammatory indicators mentioned above, multiple scholars have proposed that the ideal biomarkers for guiding and optimizing antibiotic treatment for individual patients during infection should be able to quantify pathogen load directly (Svaldi et al., 2001; Carcò et al., 2022; Shao et al., 2022; Yin et al., 2024). DdPCR for plasma pathogen cfDNA maybe this ideal choice. Multiple studies have shown that pathogen load is closely related to the severity of BSI (Lin et al., 2022; Shao et al., 2022; Lin et al., 2023; Yin et al., 2024), and ddPCR’s quintessential feature lies in its capacity to quantify pathogen concentrations within blood samples (Shao et al., 2022); it can provide more accurate information on pathogen proliferation in the bloodstream (Yin et al., 2024). Compared with conventional inflammatory markers, ddPCR is less affected by disease and immune status and provides more direct and accurate pathogen evidence. Therefore, we and previous studies agree that trend changes in pathogen DNA load detected by ddPCR can be used to dynamically monitor infection conditions to help clinicians in antimicrobial therapy stewardship in real time (Lin et al., 2022; Shao et al., 2022; Lin et al., 2023; Yin et al., 2024).

To further assess the potential clinical utility of ddPCR, we compared the TAT between ddPCR and CMT and evaluated the real-world impact of ddPCR outcomes in antimicrobial stewardship. We found that ddPCR reported pathogen results faster than CMT (7.56h vs. 19.93h, P<0.0001); this is consistent with what has been reported by Ke Lin et al (Lin et al., 2023). DdPCR could provide earlier results in 80.9% of all samples and earlier definitive pathogen evidence in 50% of patients with suspected infection by CMT.

Our ddPCR results guided 33.7% of patients to a more appropriate antibiotic regimen. An appropriate and precise antibiotic regimen for patient anti-infective treatment is very important. A study in Spain included 1615 hematologic patients with FN reported that, although 87% of cases followed the recommendations of IDSA, a total of 394 patients (24%) received inappropriate empirical antibiotic treatment (IEAT). Those who received IEAT experienced a significantly higher mortality compared to those receiving appropriate empirical antibiotic therapy (36% vs 24%, P =0.004) (Martinez-Nadal et al., 2020). Our committee evaluated the effectiveness of the antibiotic treatment regimen. Compared with empirical antibiotic treatment, ddPCR improved the effectiveness of anti-infective therapy (86.7% vs 57.6%). This improvement in effectiveness given credit to faster results, mixed infections detection, and accurate quantification of pathogen load provided by ddPCR. Another positive impact of ddPCR on therapeutic regimens is the reduction of unnecessary medications, thereby avoiding prolonged overuse of antibiotics and reducing serious complications. Therefore, we believe that ddPCR has a beneficial effect on antibiotic therapy, enabling clinics to adjust treatment plans more accurately and promptly, ultimately improving the effectiveness of antibiotic therapy for patient benefit.

There are several limitations in our study: (i) This is a single-center retrospective study with a limited number of cases, there may be deviations in the results, and multicenter studies with larger sample sizes are needed to confirm the relevant results in the future further; (ii) Currently, ddPCR can only identify a limited number of pathogens, 2 cases of pathogenic bacteria (Streptococcus mitis and Corynebacterium) identified by BC in this study were without ddPCR detection range. This indicates the limitations of ddPCR in pathogen detection due to panel design; (iii) Most microorganisms detected by ddPCR have not had the nucleic acid amplification tests for molecular verification, except for a few viruses. Molecular microbiological testing is still not widely available in developing countries, and culture and smear microscopy are still the main tools to identify pathogens (Xu et al., 2023); (iv) Discreet consideration should be given to whether the low copies pathogen results detected by ddPCR were related to clinical actual infections, the entire clinical picture must be considered when determining the clinical significance of a microbe detected by cfDNA; (v) Most patients had previous antibiotic exposure before the ddPCR test with different treatment duration and antibiotics types, the judgment of the effectiveness of antibiotic treatment may be biased, further research is needed on whether ddPCR testing improves patient prognosis.

## 5 Conclusion

Our research suggests that ddPCR has significant potential for the diagnosis and management of febrile haematological patients with suspected infections. It can enhance the ability to diagnose infections at an early clinical stage and develop appropriate antimicrobial protocols. Additionally, ddPCR can provide precise and quantitative pathogen load, dynamically monitor infection status, facilitate real-time optimization of antimicrobial regimens, and improve the effectiveness of antimicrobial therapy. However, further investigation is needed to fully explore its utility in clinical practice and prognostic management.

## Data Availability

The original contributions presented in the study are included in the article/**Supplementary Material**. Further inquiries can be directed to the corresponding author/s.
