# Supplementary material for: Plasma cell-free DNA Droplet Digital PCR provides rapid and efficient infectious microbiology diagnosis for febrile haematological patients
Source: Front Cell Infect Microbiol. 2025 Feb 19;15:1522426. doi: 10.3389/fcimb.2025.1522426 (PMC11880229; doi:10.3389/fcimb.2025.1522426)
Supplement: Supplementary file 1 [file Table1.docx]

**Supplementary Table S1** The range of pathogen detection for ddPCR

| Categories (total) | Pathogenic microorganisms | Total |
| --- | --- | --- |
| Gram-negative bacteria (G^-^) | Pseudomonas aeruginosa, Klebsiella, Escherichia coli, Acinetobacter baumannii, Burkholderia cepacia, Stenotrophomonas maltophilia, Serratia marcescens, Proteus mirabilis, Enterobacter cloacae, Citrobacter freundii, Salmonella spp., Bacteroides fragilis, Morganella morganii | 13 |
| Gram-positive bacteria (G^+^) | Staphylococcus aureus, Enterococcus spp., Streptococcus spp., Coagulase-negative Staphylococcus | 4 |
| Virus | Epstein-Barr Virus (EBV), Cytomegalovirus (CMV), Herpes simplex virus 1 (HSV-1), Herpes simplex virus 2 (HSV-2), Varicella -zoster virus (VZV) | 5 |
| Fungi | Candida spp. | 1 |

**Supplementary Table S2** Definitions

| Categories | Definitions |
| --- | --- |
| Definite | DdPCR pathogens results were consistent with at least 1 pathogen identified by BC or other microbiologic test performed within 7 days of ddPCR sample collection and was a likely cause of infectious fever. |
| Probable | DdPCR results and CMTs were discordant,ddPCR pathogen result was a likely cause of infectious fever according to composite clinical diagnosis. |
| Possible | DdPCR and CMTs were discordant, ddPCR result was consistent with an infection but not a common cause, which must be diagnosed by a combination of history and nonmicrobial testing. |
| Unlikely | DdPCR was positive but discordant with CMT and/or not a plausible cause of infection; or there was a more likely explanation for the fever event or not meeting “possible” or “probable” classification criteria. |
| False negative | DdPCR were negative,CMTs were positive and adjudicated was the cause of infection;or the ddPCR negative cases were considered composite clinical diagnosis positive. |
| True negative | DdPCR-negative result was concordant with other negative CMTs and fever was considered to a non-infectious etiology. |

**Supplementary Table S3** DdPCR results (excluding virus) versus BC positive results

| Sample Number | BC | | ddPCR | |
| --- | --- | --- | --- | --- |
|  | pathogen | TTP^a^, hours | pathogen | Copies/ml |
| 5 | Klebsiella oxytoca | 14.52 | Klebsiella | 1987.20 |
| 6 | Klebsiella oxytoca | 12.56 | Klebsiella | 10203.75 |
|  |  |  | Escherichia coli | 356.13 |
| 16 | streptococcus midis | 36.12 | Enterococcus spp. | 84.87 |
| 19 | Escherichia coli | 17.09 | Escherichia coli | 48.6 |
| 30 | Klebsiella pneumoniae | 11.52 | Klebsiella | 4607.82 |
|  |  |  | Escherichia coli | 290.16 |
| 31 | Stenotrophomonas maltophilia | 22.92 | Stenotrophomonas maltophilia | 7993.94 |
| 32 | Enterococcus faecium | 41.3 | Enterococcus spp. | 331.65 |
| 39 | Corynebacterium | 45.02 | Enterococcus spp. | 213.21 |
| 41 | Escherichia coli | 12.44 | Escherichia coli | 117.40 |
| 44 | Escherichia coli | 9.65 | Escherichia coli | 41777.05 |
| 45 | Candida tropicalis | 19.93 | Candida spp. | 436.1 |
| 46 | Candida krusei | 24.9 | negative | / |
| 48 | Candida tropicalis | 15.44 | Candida spp. | 14343.75 |
|  |  |  | Enterococcus spp. | 167.80 |
| 55 | Staphylococcus aureus | 17.18 | Staphylococcus aureus | 696.51 |
| 56 | Escherichia coli | 18.13 | Escherichia coli | 67242.4 |
| 74 | Staphylococcus epidermidis | 20.24 | negative | / |

TTP^a^: Time to positive.
